# Immunomodulatory and Inhibitory Effect of Immulina^®^, and Immunloges^®^ in the Ig-E Mediated Activation of RBL-2H3 Cells. A New Role in Allergic Inflammatory Responses

**DOI:** 10.3390/plants7010013

**Published:** 2018-02-26

**Authors:** Kurt Appel, Eduardo Munoz, Carmen Navarrete, Cristina Cruz-Teno, Andreas Biller, Eva Thiemann

**Affiliations:** 1 VivaCell Biotechnology GmbH, Denzlingen 79211, Germany; appel@vivacell.de; 2 VivaCell Biotechnology España, Córdoba 14004, Spain; e.munoz@uco.es (E.M.); c.navarrete@vivacellspain.com (C.N.); c.cruz@vivacellspain.com (C.C.-T.); 3 Dr. Loges + Co. GmbH, Winsen 21423, Germany; biller@loges.de

**Keywords:** allergy, inflammation, mast cells, Immulina^®^, immunLoges^®^

## Abstract

Immulina^®^, a high-molecular-weight polysaccharide extract from the cyanobacterium *Arthrospira platensis* (*Spirulina*) is a potent activator of innate immune cells. On the other hand, it is well documented that *Spirulina* exerts anti-inflammatory effects and showed promising effects with respect to the relief of allergic rhinitis symptoms. Taking into account these findings, we decided to elucidate whether Immulina^®^, and immunLoges^®^ (a commercial available multicomponent nutraceutical with Immulina^®^ as a main ingredient) beyond immune-enhancing effects, might also exert inhibitory effects in the induced allergic inflammatory response and on histamine release from RBL-2H3 mast cells. Our findings show that Immulina^®^ and immunLoges^®^ inhibited the IgE-antigen complex-induced production of TNF-α, IL-4, leukotrienes and histamine. The compound 48/80 stimulated histamine release in RBL-2H3 cells was also inhibited. Taken together, our results showed that Immulina^®^ and immunLoges^®^ exhibit anti-inflammatory properties and inhibited the release of histamine from mast cells.

## 1. Introduction

*Arthrospira platensis* (*Spirulina*) is a microscopic and filamentous cyanobacterium. It has a long tradition of use as food and has been used by humans for many years as food supplement in the form of health drinks, pills, or tablets. *Spirulina* became famous after it was used by NASA as a dietary supplement for astronauts. The safety of Spirulina has been well established. The Food and Drug Administration (FDA) has categorized *Arthrospira* products “Generally Recognized as Safe” (GRAS). *Spirulina* has high nutritional values due to its high content in proteins (up to 70%), essential amino acids, minerals, essential fatty acids, vitamins, and antioxidants [1]. Apart from high nutritional values *Spirulina* showed hypolipidemic, hypoglycemic, antihypertensive properties, microbial-modulating, antioxidant and anti-inflammatory activities [2]. In addition, *Spirulina* may be useful for the treatment of allergic conditions [3,4,5]. A prospective study found a high prevalence of *Spirulina* usage for the relief of allergic rhinitis symptoms in Turkey [6]. On the other hand, polysaccharides contained in *Spirulina* also have immunostimulatory activity. The main active compounds within Immulina^®^, a commercial extract of *Spirulina*, are bacterial Braun-type lipoproteins that activate innate immune cells through a toll-like receptor (TLR) 2-dependent pathway [7]. Previous studies indicate that Immulina^®^ is a potent in vitro [8,9] and in vivo [10,11] immune cell activator. In addition, Immulina^®^ can exhibit a protective effect against influenza A (H1N1) viral infection [12]. Immulina^®^ which has been commercialized as a dietary supplement for modulating immune function, as well as ameliorating various diseases, is also an ingredient of multicomponent-nutraceuticals e.g., of immunLoges^®^. 

Macrophages are an important component of the innate immune system and clear organisms through their phagocytic function. Toll-like receptors expressed by macrophages play an important role in their activation. Macrophages at tissue sites of allergic inflammation originate from mononuclear cells in the bone marrow. In addition to their phagocytic and antigen presenting role, macrophages have the potential to be proinflammatory or anti-inflammatory based on the spectrum of mediators they can release. There are two major pathways of the macrophage activation required for their main functional activities: the classical M1 pathway and the alternative M2 pathway [13,14]. The classical pathway gives rise to the M1 macrophages during an immune response to infection and is mediated by the Th1 cytokine interferon gamma (IFN-γ) and the toll-like receptor agonists, such as lipopolysaccharide (LPS). IL-17 also modulates the differentiation of macrophages to the pro-inflammatory M1 phenotype [15]. The classically activated M1 macrophages express and secrete large amounts of potent pro-inflammatory mediators such as tumor necrosis factor (TNF) and reactive oxygen species. Moreover, the M1 macrophages express high levels of major histocompatibility complex (MHC) class II and, co-stimulatory molecules, such as CD86, which are important for the activation and stimulation of CD4 T cells. The alternatively activated M2 macrophages are induced by the Th2-derived cytokines IL-4 or IL-13 (IL-4/IL-13-induced phenotype is also called the “M2a” subset of M2 population). The M2 macrophages produce a number of factors that are responsible for the anti-parasitic response and promote tissue repair such as Fizz1, Ym1, Arg1, Mannose Receptor (MR or CD206) as well as anti-inflammatory, regulatory, and B cell-stimulating factors such as TGF-β1, IL-4, and IL-10 [14,16].

Allergic airway diseases, such as asthma and allergic rhinitis, are common diseases caused by hypersensitivity of the immune system. Approximately 10–20% of the world population is affected by allergies, with the number of allergy patients increasing annually [17,18]. Most allergy patients are genetically predisposed to produce IgE. Mast cells play an important role in innate and adaptive immunity, especially in allergic and inflammatory responses. Mast cells express a high-affinity IgE receptor on membranes, and the binding of IgE-antigen complexes to the FcεRI (high-affinity receptor for the Fc region of immunoglobulin E) receptor triggers complex biological reactions. Activation of rat basophilic leukemia cells (RBL-2H3) cells by an IgE-antigen complex induced the production of TNF-alpha and IL-4. Mast cells are a key player in early allergic response, which typically occurs within minutes of exposure to an appropriate antigen, and other biological responses, including inflammatory disorders [19]. These cells are critical effector cells in IgE-dependent immediate hypersensitivity reactions [20]. Mast cell degranulation can initiate an acute inflammatory response and contribute to the progression of chronic diseases [21]. When an IgE-antigen binds with FcεRI, the receptor is activated, and a variety of biologically active mediators are released, causing allergic reactions, including the release of histamine, arachidonic acid metabolites, and inflammatory cytokines [22]. Importantly, arachidonic acid metabolites, including prostaglandins and leukotrienes, mediate acute and chronic allergic reactions [23,24]. RBL-2H3 cells are a mast cell line that originated from rat basophilic leukemia and have been widely used to study IgE-FcεRI interactions and degranulation. Furthermore, RBL-2H3 cells are a useful model for in vitro screening of antiallergy drug candidates.

The aim of the present study was to determine whether Immulina^®^ despite its well documented immunostimulatory activity and/or immunLoges^®^, also have anti-inflammatory activities and may be useful for the treatment of allergic conditions.

## 2. Results

### 2.1. Cytotoxicity Effects of Immunloges^®^ and Immulina^®^ in Raw264.7 Cells

**Figure 1:**
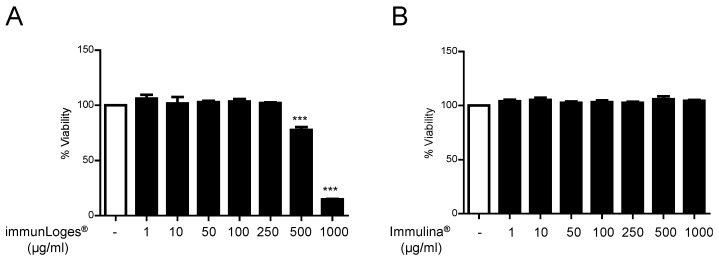
Cytotoxic activity of immunLoges^®^ and Immulina^®^ in Raw264.7 cells. The cells were treated with immunLoges^®^ (**A**) or Immulina^®^ (**B**) at the indicated concentrations for 24 h. Cytotoxicity was measured by fluorescence microscopy using an Incucyte System. Data represent the mean ± SD (*n* = 3). *** *p* ˂ 0.001 vs. untreated. (one-way analysis of variance (ANOVA) followed Newman-Keuls test).

To analyze the potential cytotoxic activities of the test substances Raw264.7 cells were incubated with increasing concentrations of immunLoges^®^ and Immulina^®^ for 24 and the cytotoxicity was measured by YOYO-1 staining and fluorescence microscopy using an Incucyte System. Only immunLoges**^®^** at the highest concentration tested (1000 µg/mL) showed cytotoxicity at 24 h (Figure 1). After analyzing the potential cytotoxicity in primary cells, it was decided to use the concentrations described in Material and Methods.

### 2.2. NF-ĸB Activation in Raw264.7 Cells

**Figure 2:**
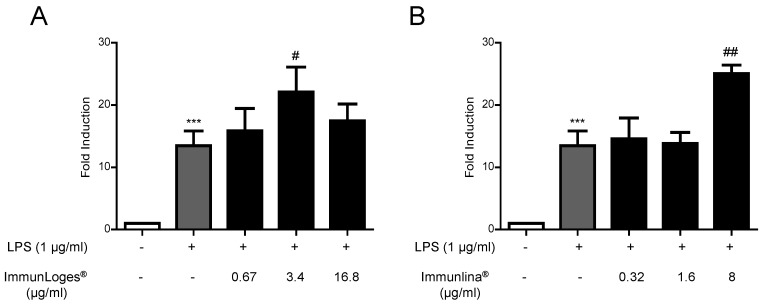
NF-ĸB activity of immunLoges^®^ (**A**) and Immulina^®^ (**B**) in Raw264.7-KBF-Luc cells. Cells were pre-incubated with the test substances at the indicated concentrations for 30 min and then stimulated with LPS for 6 h. The results of the specific transactivation are expressed as a fold induction over untreated cells. Data represent the mean ± SD (*n* = 3). *** *p* ˂ 0.001 vs. untreated, # *p* ˂ 0.05, ## *p* ˂ 0.01 vs. LPS treatment. (one-way ANOVA followed Newman-Keuls test).

Although previous studies indicate that Immulina^®^ is a potent in vitro and in vivo immune cell activator, we wanted to know the activity of the commercial extract immunLoges^®^. In this sense we evaluated the effect of the active component and the commercial extract on the NF-κB transcriptional activity. This activity was evaluated by using the luciferase reporter construct KBF-Luc [25]. Activation by LPS clearly increased (13-fold induction) the luciferase gene expression driven by the NF-κB dependent promoter in stably transfected Raw264.7 cells. We found that, immunLoges^®^ and Immulina^®^ increase this activity (Figure 2A,B).

### 2.3. Effect of Immunloges^®^ and Immulina^®^ on M1 and M2 Polarization

Macrophages are also important effector cells that mediate the immune responses. They act as antigen presenting cells (APC), thereby activating an antigen-specific T cell response in the periphery and central nervous system (CNS). Macrophages detect the endogenous danger signals that are present in the debris of necrotic cells through Toll-like receptors (TLRs) 2,6,intracellular pattern-recognition receptors and the interleukin-1 receptor (IL-1R), most of which signal through the adaptor molecule myeloid differentiation primary-response gene 88 (MyD88). This function makes macrophages one of the primary sensors of danger in the host. 

**Figure 3:**
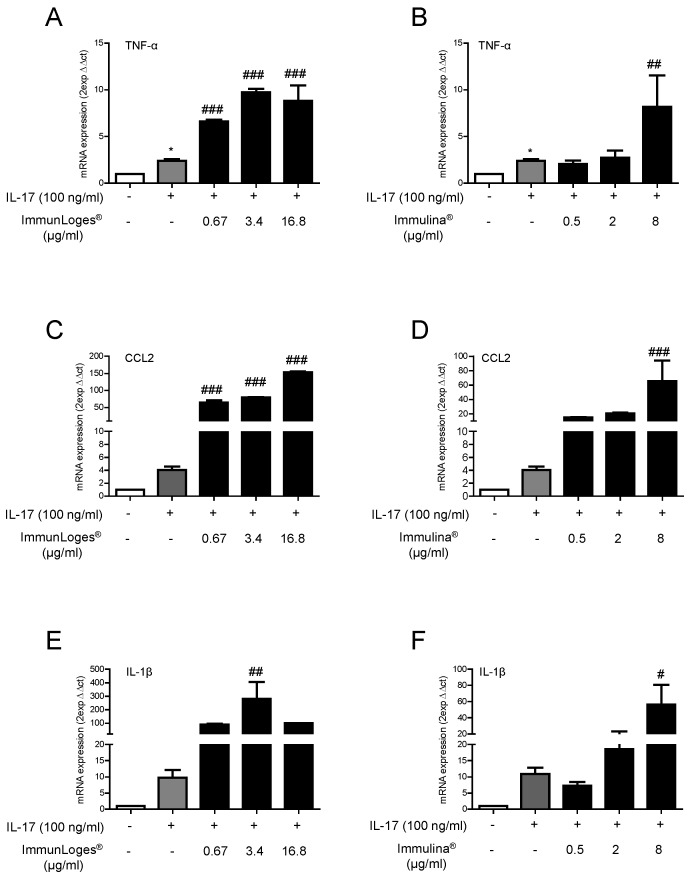
Effect of immunLoges^®^, Immulina^®^ on IL-17-induced M1 markers expression. Cells were pre-incubated with immunLoges^®^ or Immulina^®^ at the indicated concentrations for 18 h and then stimulated with IL-17 for 24 h. TNF-α (**A**,**B**), CCL2 (**C**,**D**) and IL-1β (**E**,**F**) expression was determined. The GAPDH gene was used to standardize mRNA expression in each sample and gene expression was quantified using the 2-ΔΔCt method. Data represent the mean ± SD (*n* = 3). * *p* ˂ 0.05 vs. untreated, # *p* ˂ 0.05, ## *p* ˂ 0.01, ### *p* ˂ 0.001 vs. IL-17 treatment. (one-way ANOVA followed Newman-Keuls test.

Treatment of RAW264.7 macrophages with IL-17 promoted their polarization towards a pro-inflammatory M1 phenotype, as shown by increased expression of M1 markers such as TNF-α, CCL2 or IL-1β. Raw264.7 cells were pre-treated for 18 h with the test substances and then were exposed to recombinant murine IL-17 to induce M1 polarization and M1 markers were analyzed by qPCR. The treatment with immunLoges^®^ and Immulina^®^ clearly enhanced the expression of IL17-induced M1 markers TNF-α, CCL2 and IL-1ß as shown in Figure 3.

**Figure 4:**
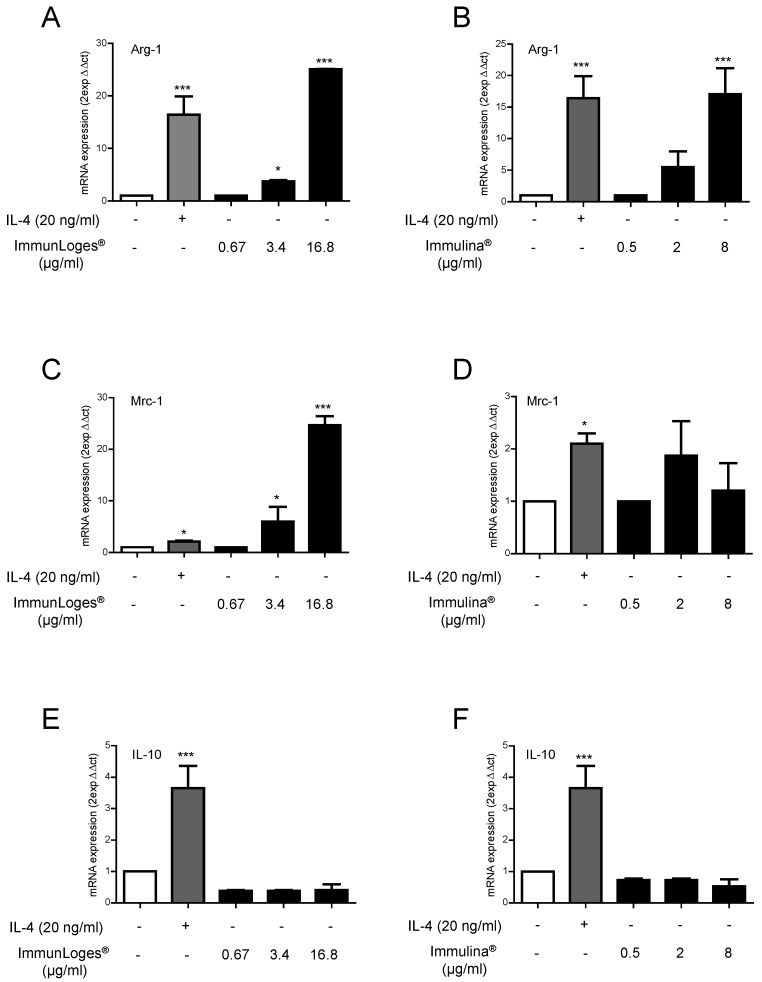
Effect of immunLoges^®^, Immulina^®^ on M2 polarization. Cells were pre-incubated with immunLoges^®^ or Immulina^®^ at the indicated concentrations for 24 h. Arg-1 (**A**,**B**), Mrc-1 (**C**,**D**) and IL-10 (**E**,**F**) expression was determined. The GAPDH gene was used to standardize mRNA expression in each sample and gene expression was quantified using the 2-ΔΔCt method. Data represent the mean ± SD (*n* = 3). * *p* ˂ 0.05, *** *p* ˂ 0.001 vs. untreated. (one-way ANOVA followed Newman-Keuls test).

To study the effect of immunLoges^®^ and Immulina^®^ on M2 polarization, Raw264.7 cells were treated for 24 h with the test substances at the indicated concentrations. As positive control we used the recombinant mouse IL-4 to induce M2 polarization. M2 markers such as Arg1, Mrc1 and IL-10 were determined. The treatment with immunLoges^®^ and Immulina^®^ clearly induced the expression of the M2 markers Arg1 (Figure 4A,B), and Mrc1 (Figure 4C,D). In the case of IL10 gene expression, the treatment did not impair the gene expression (Figure 4E,F).

### 2.4. Cytotoxicity Effects of Immunloges^®^ and Immulina^®^ in RBL-2H3 Cells

**Figure 5:**
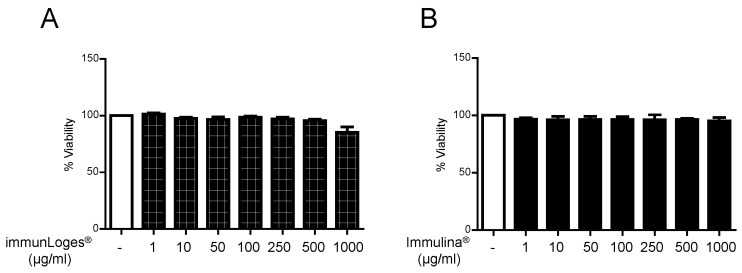
Cytotoxic activity of immunLoges^®^ (**A**) and Immulina^®^ (**B**) in RBL-2H3 cells. The cells were treated with immunLoges^®^ or Immulina^®^ at the indicated concentrations for 24 h. Cytotoxicity was measured by fluorescence microscopy using an Incucyte System. Data represent the mean ± SD (*n* = 3).

To evaluate the cytotoxicity of immunLoges^®^ and Immulina^®^ on RBL-2H3, the cells were treated with different concentrations for 24 h and the cytotoxicity was measured by fluorescence microscopy using an Incucyte System. Neither Immulina^®^ nor immunLoges^®^ showed cytotoxicity at the concentrations investigated in this study (Figure 5).

### 2.5. Effect on Cytokines Release in IgE-Antigen Complex Stimulated RBL-2H3 Cells

Mast cells express a high-affinity IgE receptor (FcεRI) on membranes, and the binding of IgE-antigen complexes to FcεRI triggers complex biological reactions. RBL-2H3 cells were sensitized with anti-DNP-IgE (monoclonal anti-dinotrophenyl antibody produced in mouse, IgE isotype) and stimulated with DNP-HSA (dinitrophenyl-human serum albumin). Inflammatory cytokines mediate the pathological reaction in inflammatory diseases. Besides the secretion of stored cytokines during degranulation, the production of newly generated inflammatory cytokines (TNF-α, IL-4, IL-6 etc.) is induced.

**Figure 6:**
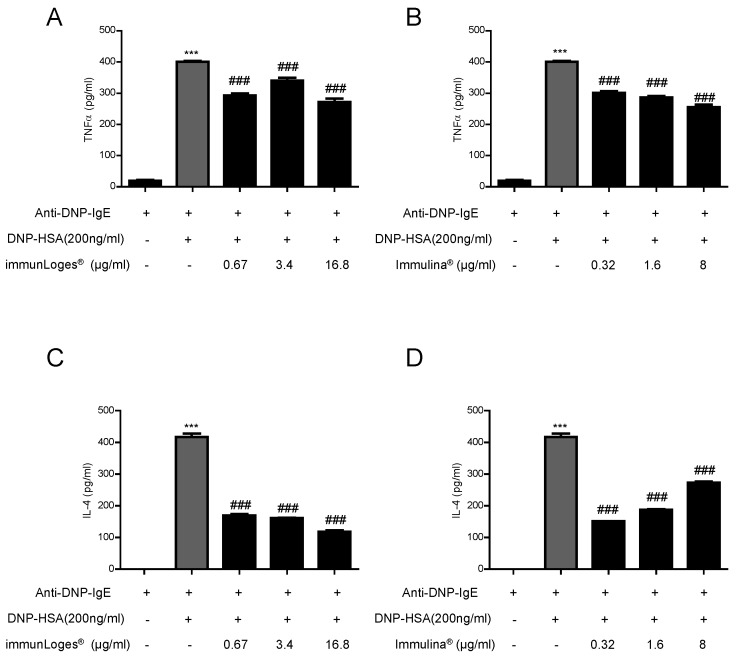
Effect of immunLoges^®^ and Immulina^®^ on TNF-α and IL-4 production in IgE-antigen complex-stimulated RBL-2H3 cells. The cells were treated with immunLoges^®^ (**A**,**C**) or Immulina^®^ (**B**,**D**) at the indicated concentrations and the levels of TNF-α and IL-4 were determined in the supernatants. Data represent the mean ± SD (*n* = 3). *** *p* ˂ 0.001 vs. Anti-DNP-IgE, ### *p* ˂ 0.001 vs. Anti-DNP-IgE+DNP-HAS treatment. (one-way ANOVA followed Newman-Keuls test).

The effects of the test substances on pro-inflammatory cytokine production were assessed by ELISA. Activation of RBL-2H3 cells by an IgE-antigen complex induced the production of TNF-α and IL-4. The treatment with immunLoges^®^ (Figure 6A) or Immulina^®^ (Figure 6B) decreased the levels of TNF-α and IL-4 (Figure 6C,D) in FcεRI-activated RBL-2H3 cells.

### 2.6. Effect on Leukotrienes Production in IgE-Antigen Complex-Stimulated RBL-2H3 Cells

**Figure 7:**
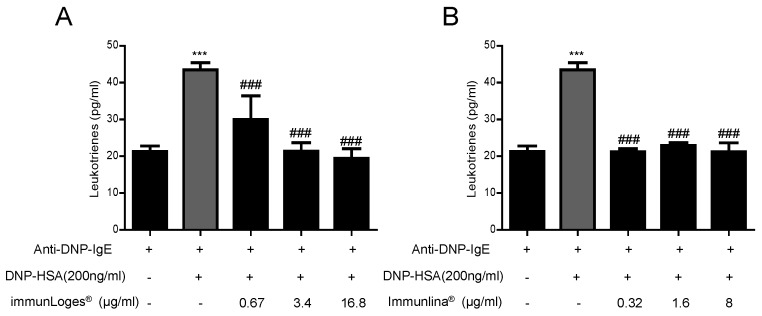
Effect of immunLoges^®^ and Immulina^®^ on leukotrienes production in IgE-antigen complex-stimulated RBL-2H3 cells. The cells were treated with immunLoges^®^ (**A**) and Immulina^®^ (**B**) at the indicated concentrations and the levels of leukotrienes were determined in the supernatants. Data represent the mean ± SD (*n* = 3). *** *p* ˂ 0.001 vs. Anti-DNP-IgE, ### *p* ˂ 0.001 vs. Anti-DNP-IgE+DNP-HSA treatment. (one-way ANOVA followed Newman-Keuls test).

In parallel to the analysis of cytokine production we determined the formation of leukotrienes LTC4/D4/E4 in supernatants of IgE-antigen complex-stimulated RBL-2H3 cells. The treatment with immunLoges^®^ (Figure 7A) and Immulina^®^ (Figure 7B) reduced the levels of leukotrienes.

### 2.7. Effect on the Release of Histamine in IgE-Antigen Complex-Stimulated RBL-2H3 Cells

**Figure 8:**
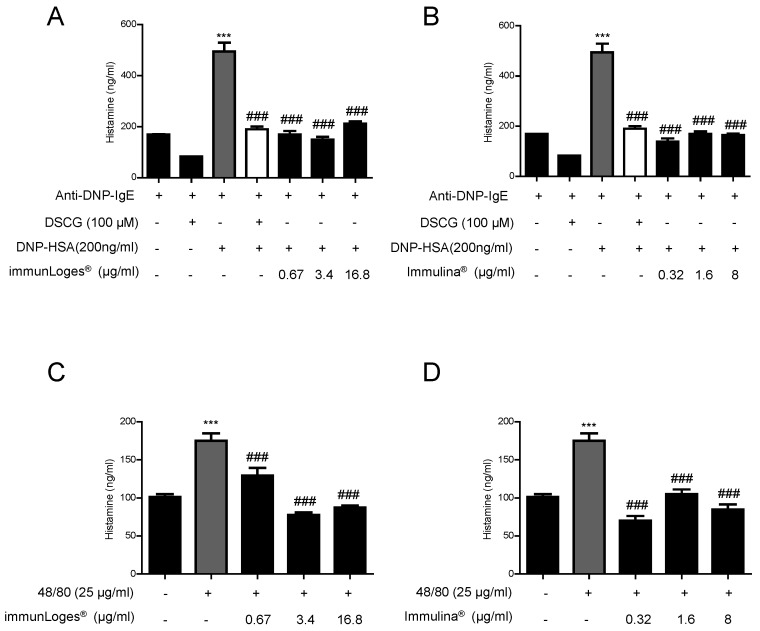
Effect of immunLoges^®^ and Immulina^®^ on mast stabilization. The cells were pretreated for 2 h with immunLoges^®^ (**A**,**C**) and Immulina^®^ (**B**,**D**) at the indicated concentrations and stimulated as indicated. The levels of histamine were determined in the supernatants. Data represent the mean ± SD (*n* = 3). *** *p* ˂ 0.001 vs. Anti-DNP-IgE or untreated cells, ### *p* ˂ 0.001 vs. Anti-DNP-IgE+DNP-HAS or 48/80 treatment. (one-way ANOVA followed Newman-Keuls test).

Optimum conditions for IgE mediated degranulation in RBL-2H3 cells were sensitizing with anti-DNP-IgE and stimulating with 2,4-Dinitrophenyl hapten conjugated to human serum albumin (DNP-HSA). The release of histamine in IgE-antigen complex-stimulated RBL-2H3 cells were significantly increased compared with untreated cells. Pre-treatment for 2 h with the test substances at the indicated concentrations clearly suppressed the degranulation in IgE-antigen complex-stimulated RBL-2H3 cells. In addition, we tested the mast cell stabilizing agent Disodium cromoglycate (DSCG). As it is shown in Figure 8 the treatment with this compound inhibited the degranulation in a similar manner to that observed for immunLoges^®^ (Figure 8A) and Immulina^®^ (Figure 8B).

The effects of synthetic compound 48/80 were also examined, since earlier studies have shown that this compound is a very potent inducer of mast cell degranulation. Compound 48/80 is a G protein stimulant compound originally described as a mast cell secretagogue. Pre-treatment for 2 h with the compounds at the indicated concentrations inhibited the release of histamine from RBL-2H3 stimulated by compound 48/80 (Figure 8 C,D).

**Figure 9:**
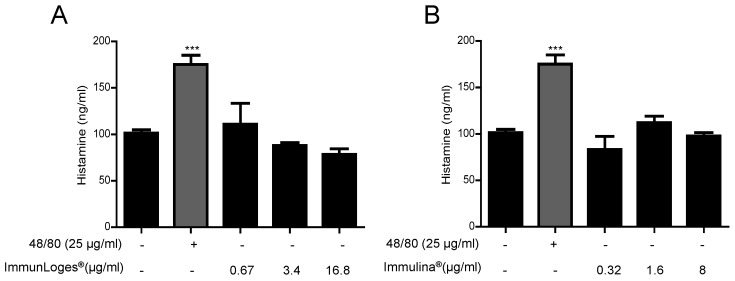
Effect of immunLoges^®^ and Immulina^®^ on mast stabilization. The cells were treated for 2 h with immunLoges^®^ (**A**) and Immulina^®^ (**B**) at the indicated concentrations. The levels of histamine were determined in the supernatants. Data represent the mean ± SD (*n* = 3). *** *p* ˂ 0.001 vs. untreated cells (one-way ANOVA followed Newman-Keuls test).

Finally, we determined the effect of immunLoges^®^ and Immulina^®^ on the basal release of histamine in the mast cell line. The treatment with the test substances at the concentrations investigated did not induce histamine secretion (Figure 9).

## 3. Discussion

Previous reports showed that Immulina^®^ is a potent immune cell activator and activates NF-ĸB through TLR2 receptor [7,8]. Here we describe the activation of NF-ĸB in macrophages treated with immunLoges^®^ and Immulina^®^ in the presence of LPS confirming those previous studies. Herein we show that immunLoges^®^ preserves the immunomodulatory activity.

Traditionally, macrophages have been seen only as immune cells, and their homeostatic responsibilities have been overlooked. Mosser et al. [26], proposed that macrophages exist in three main categories: host defense, wound healing, and immune regulation. M1 macrophages (classically activated) clears tissue of cellular debris and pathogens via aggressive phagocytosis and release of pro-inflammatory cytokines including: IL-1, IL-6, IL-12, IL-23, and TNF-α. The M2 macrophage acts as a support cell by promoting the healing of damaged cells by angiogenesis and tissue remodeling via secretion of anti-inflammatory cytokines. While these two operationally useful polarities exist, it is widely believed that macrophages exist mainly in the continuum between these two phenotypes [27]. Regulation of this continuum involves complex processes that have been extensively studied, yet remain relatively unclear. Recently it was shown that macrophages will respond to injury or infection by upregulating pro-inflammatory (M1) responses, but later switch to an anti-inflammatory (M2) response once the infection is under control [28,29]. This M1 activation program is typically associated with protection against disease, and M1 polarization has been shown to aid host control of several bacteria, including Listeria monocytogenes, Salmonella typhimurium, Mycobacterium tuberculosis and Chlamydia infections [30,31,32].

Here we describe a dual role for immunLoges^®^ and Immulina^®^ in the macrophage polarization. On the one hand, we observed an increase of M1 markers such as TNF-α, CCL2 and IL-1β in the presence of LPS. This effect on M1 polarization is important from the point of view of the common response of macrophages to bacterial infections which mainly involve the up-regulation of genes involved in M1 polarization. Moreover, other M1 associated up-regulated genes encode the enzymes indoleamine-pyrrole 2,3 dioxygenase and NO synthase 2 (NOS2), which are related with macrophage microbicidal activity [30]. The effect of immunLoges^®^ and Immulina^®^ on M2 polarization could be related with immunoregulatory activity since M2 macrophages dampen inflammation, suppress antitumor immunity and promote wound healing and tissue remodeling.

Although more sophisticated mechanisms are still unknown, data from animal studies suggest that mast cells act similarly to macrophages and other inflammatory cells and contribute to human diseases through cell–cell interactions and the release of proinflammatory cytokines, chemokines, and proteases to induce inflammatory cell recruitment. There is growing interest in understanding how mast cells participate in positive immunoregulation particularly in the innate aspects of an immune response [33,34,35].

Mast cells are important in innate immune system. They have been appreciated as potent contributors to allergic reaction. Mast cells have a widespread distribution and are found predominantly at the interface between the host and the external environment and acted as both first responders in harmful situations as well as to respond to changes in their environment by communicating with a variety of other cells implicated in immunological responses. Therefore, the critical role of mast cells in both innate and adaptive immunity, including immune tolerance, has gained increased prominence [36,37,38]. Conversely, mast cell dysfunction has pointed to these cells as the main offenders in several chronic allergic/inflammatory disorders, cancer, and autoimmune diseases [39,40,41]. 

Moreover, it has been well documented that *Spirulina* exhibits anti-inflammatory properties by inhibiting the release of histamine from mast cells [3]. In a randomized double-blind placebo-controlled trial individuals with allergic rhinitis *Spirulina* significantly reduced the levels of IL-4 that is important in regulating immunoglobulin (Ig)E-mediated allergy [5].

Since high molecular weight polysaccharides are present in Immulina^®^ it is possible that different preparations of these algae may exert different immunological activities. Immulina^®^ and immunLoges^®^ decreased the levels of TNF-α and IL-4 in FcεRI-activated RBL-2H3 cells. These results indicated the anti-allergic property of both substances in allergic inflammation therapy.

In parallel to the analysis of cytokine production we tested the release of leukotrienes, which are important mediators both in host defense mechanisms and in inflammatory disease states. Leukotrienes can be formed in mast cells, granulocytes, and monocytes/macrophages in response to extracellular stimulation. In this study, we determined the formation of leukotrienes LTC4/D4/E4 in supernatants of IgE-antigen complex-stimulated RBL-2H3 cells. The treatment with Immulina^®^ reduced the levels of leukotrienes.

Apart from the anti-inflammatory parameters we also investigated the effects on mast cell stabilization and histamine release. Immulina^®^ inhibited the release of histamine in IgE-antigen complex-stimulated RBL-2H3 cells in a similar manner to that observed for the mast cell stabilizing agent Disodium cromoglycate (DSCG). In addition, we determined the effect of the test substances on the secretion of histamine induced by compound 48/80. This G protein stimulant compound was originally described as a mast cell secretagogue. Again, Immulina^®^ inhibited the compound 48/80 stimulated histamine release in RBL-2H3 cells.

In parallel we tested immunLoges^®^ in the above-mentioned assays and immunLoges^®^ showed similar effects compared to Immulina^®^. Therefore, neither additional compounds (vitamins etc.) nor excipients in immunLoges^®^ interfered with the described activities.

Immulina^®^ is a patented, high molecular weight polysaccharide extract from *spirulina* and the predominant active compounds are Braun-type lipoproteins. Although Immulina^®^ is a potent NF-ĸB and immune cell activator, our results clearly showed that Immulina^®^ has anti-inflammatory and anti-histaminic effects in RBL-2H3 cells. 

In general, the results obtained for both products, Immulina^®^ and immunLoges^®^, are dose-response independent, which can be explained by the fact that most of the studies for both compounds had strong activity at the lowest concentrations studied.

Overall, our results with Immulina^®^ and immunLoges^®^ agree with the concept that *Spirulina* exerts anti-inflammatory effects and may be useful for the treatment of allergic conditions, but further in vivo studies should be performed to clarify these effects for Immulina^®^ and immunLoges^®^.

## 4. Materials and Methods 

### 4.1. Test items

Immulina^®^ is a patented extract [22] derived from *Arthrospira platensis* (Nordstedt) Gomont, Phormidiaceae (previously called *Spirulina platensis* and commonly known as *Spirulina*). This extract represents a 10–15% yield from spray dried *Arthrospira platensis* and is standardized by biological activity to ensure consistent activity of the extract from batch to batch. Biological standardization is performed using a humanTHP-1 monocyte activation assay that measures activity as indicated by increased expression of an NF-kB-driven luciferase reporter as previously described (Pugh et al. 2001). The Immulina^®^ extract (batch number 600137) used in this research was supplied by ChromaDex Inc. (Irvine, CA, USA). immunLoges^®^ (batch 500875, Dr. Loges + Co. GmbH, Winsen, Germany) is a multi-complex formulation of Immulina^®^, Betox 93^®^, Selenium, Zink, Vitamin C, D and resveratrol. One tablet immunLoges^®^ (420 mg) contains among others 200 mg Immulina^®^. The composition of commercial extract for ImmunLoges^®^ is described in the Table 1 below. For a better comparison of the results, we tested the immunLoges^®^ concentration which correspond to the Immulina^®^ concentration in immunLoges^®^. The stock solutions were prepared in DMSO and then diluted in cell culture media. The final DMSO concentration never exceeded 0.1% (*v*/*v*).

### 4.2. Cell Culture

RAW264.7 mouse macrophage cell line was obtained from ATCC (TIB-71) and maintained at 37 °C in a humidified atmosphere containing 5% CO_2_ in (Dulbecco’s Modified Eagle Medium) (DMEM) supplemented with 10% fetal calf serum (FCS), 2 mM L-glutamine and 1% (*v*/*v*) penicillin/streptomycin. Raw264.7-kappaB Factor (KBF)-Luc cell line is stably transfected cell line with the KBF-Luc plasmid that contains the luciferase gene driven by an artificial promoter made by 3 multimerized copies of the NF-kB binding site from the MHCI Class I promoter. These cells were maintained in DMEM medium supplemented with antibiotics and 10% heat-inactivated FBS. The rat basophilic leukemia cell line RBL-2H3 was obtained from ATCC (CRL-2256) and grown in Dulbecco’s modified Eagle medium with 100 IU/mL penicillin, 100 mg/mL streptomycin, and 10% heat-inactivated fetal calf serum (FCS) in a humidified 5% CO_2_ atmosphere at 37 °C. 

### 4.3. Cytotoxicity

To identify non-cytotoxic concentrations of the test items RAW264.7 or RBL-2H3 cells were treated with increasing concentrations of the test compounds for 24 h. Cytotoxicity was measured by fluorescence (Essen BioScience, Inc., Ann Arbor, MI, USA) using YOYO-1, purchased from Thermo Fisher Scientific (Waltham, MA, USA), is a green fluorescent dye used in DNA staining. The dye was diluted in cell cultured medium and added to a final concentration of 0.1 µM. 

After 3–4 h the total number of DNA containing objects was measured by treating cells with 0.0625% triton X-100 to permeabilize the cell membrane. Object counting analysis was performed using the Incucyte FLR software (Essen BioScience, Inc., Ann Arbor, MI, USA) to calculate the total number of YOYO-1 fluorescence positive cells and total DNA containing objects (end point). The cytotoxicity index was calculated by dividing the number of YOYO-1 fluorescence positive objects by the total number of DNA containing objects for each treatment group.

### 4.4. NF-ĸB Activation in Raw264.7-KBF-Luc Cells

To study the potential NF-ĸB modulatory activity of the test items we used the stably transfected Raw264.7-KBF-Luc cell line. Cells were pretreated with the test substances for 30 min and then were stimulated with LPS for 6 h. In addition, cells were treated with the test compounds in the absence of LPS. Then the cells were washed and lysed in 25 mM Tris-phosphate pH 7.8, 8 mM MgCl2, 1 mM DTT, 1% Triton X-100, and 7% glycerol. Luciferase activity was measured using an Autolumat LB 953 (EG&G Berthold, Oak Ridge, TN, USA) following the instructions of the luciferase assay kit (Promega, Madison, WI, USA). The results of the specific transactivation are expressed as a fold induction over untreated cells.

### 4.5. IL-17-Induced M1 Polarization

Serum-starved Raw264.7 macrophages were pre-incubated with increasing concentrations of the test compounds for 18 h, before an additional 24-h treatment with the recombinant mouse IL-17 (100 ng/mL). Then cells were collected and total RNA extracted using the High Pure RNA Isolation kit (Roche Diagnostics, Hong Kong, China). Total RNA (1 µg) was retrotranscribed using the iScriptTM cDNA Synthesis Kit (Bio-Rad; Hercules, CA, USA), and the cDNA generated analyzed by real-time PCR, using the iQTM SYBR Green Supermix (Bio-Rad; Hercules, CA, USA). Real-time PCR was performed using a CFX96 Real-time PCR Detection System (Bio-Rad; Hercules, CA, USA). The GAPDH gene was used to standardize mRNA expression in each sample and gene expression was quantified using the 2-ΔΔCt method. The markers analyzed for M1 polarization were TNF-α, CCL2 and IL-1ß.

### 4.6. IL-4-Induced M2 Polarization

Serum-starved Raw264.7 macrophages were pre-incubated with increasing concentrations of the test compounds for 24 h. As positive control for M2 polarization we treated the cells with the recombinant mouse IL-4 (20 ng/mL) for 24 h. Then cells were collected and total RNA extracted using the High Pure RNA Isolation kit (Roche Diagnostics, Hong Kong, China). Total RNA (1 µg) was retrotranscribed using the iScriptTM cDNA Synthesis Kit (Bio-Rad; Hercules, CA, USA), and the cDNA generated analyzed by real-time PCR, using the iQTM SYBR Green Supermix (Bio-Rad; Hercules, CA, USA). Real-time PCR was performed using a CFX96 Real-time PCR Detection System (Bio-Rad; Hercules, CA, USA). The GAPDH gene was used to standardize mRNA expression in each sample and gene expression was quantified using the 2-ΔΔCt method. The markers analyzed for M2 polarization were Arg1, IL-10 and Mrc1.

### 4.7. Detection of Leukotrienes and Cytokines in RBL-2H3 Cells

RBL-2H3 cells were seeded at 2 × 10^5^ cells in 24-well plates and after 24 h cells were incubated overnight with or without anti-DNP IgE mAb (Santa Cruz Biotechnology, Dallas, TX, USA) (0.5 µg/mL) for sensitization. Next, cells were pre-incubated with the test substances for 1 h and then stimulated with DNP-HSA (Santa Cruz Biotechnology) (200 ng/mL**)** for 3 h. Supernatants were collected, centrifuged for 10 min at 400× *g* (4 °C) and the leukotrienes (LTC4/D4/E4), IL-4 and TNF-α were analyzed. Leukotrienes kit was obtained from Enzo Life Sciences (New York, NY, USA) IL-4 and TNF-α ELISA kits were purchased from R&D Systems (Minneapolis, MN, USA). The levels of leukotrienes and cytokines were analyzed with plate-bound ELISA kits according to the manufacturer’s recommendations. 

### 4.8. Detection of Histamine Release in RBL-2H3 Cells

RBL-2H3 cells were seeded at 2 × 10^5^ cells in 24-well plates and after 24 h the medium in each well was aspirated and replaced by fresh medium. Next cells were pre-incubated with the compounds for 2 h and then were stimulated with the compound 48/80 (25 μg/mL) (Sigma Aldrich, St. Louis, MO, USA) in the presence or absence of non-cytotoxic concentrations of the test substances for 15 min. After treatment, supernatants were collected and centrifuged for 10 min at 400× *g* (4 °C). Histamine Elisa Fast Track was purchased from LDN GmbH, Germany. Histamine in the supernatant was determined by an ELISA assay according to the manufacturer’s recommendations.

### 4.9. RBL-2H3 Mast Cells Stabilization

RBL-2H3 cells were seeded at 2 × 10^5^ cells in 24-well plates and after 24 h cells were incubated overnight with or without anti-DNP IgE mAb (0.5 µg/mL) for sensitization. Next, cells were pre-incubated with the test substances or the well-known mast stabilizing agent Disodium cromoglycate (DSCG) for 2 h and then stimulated with DNP-HSA (1 μg/mL**)** for 30 min. Supernatants were collected, centrifuged for 10 min at 400× *g* (4 °C) and histamine release was determined as above described.

### 4.10. Statistical Analysis

Data were analyzed by one-way analysis of variance (ANOVA) followed by Newman Keuls post-hoc test using GraphPad Prism version 6.00 (GraphPad, San Diego, CA, USA). Results were presented as means ± SD and differences were considered significant when *p* < 0.05.

## Figures and Tables

**Table 1:** Composition of commercial extract immunLoges^®^. The composition of immunLoges^®^ per capsule is indicated in the table. Excipients (54.5 mg) such as cellulose, talcum and magnesium.

Composition	Per Capsule
Immulina^®^	200 mg
Betox 93^®^	60 mg
Vineatrol^®^	0.5 mg
Vitamin C	40 mg
Vitamin D	10 µg
Selenite	50 µg
Zinc	5 mg
